# Supplementary figures and images for: Ambivalent partnership of the Drosophila posterior class Hox protein Abdominal-B with Extradenticle and Homothorax
Source: PLoS Genet. 2025 Jan 13;21(1):e1011355. doi: 10.1371/journal.pgen.1011355 (PMC11759358; doi:10.1371/journal.pgen.1011355)

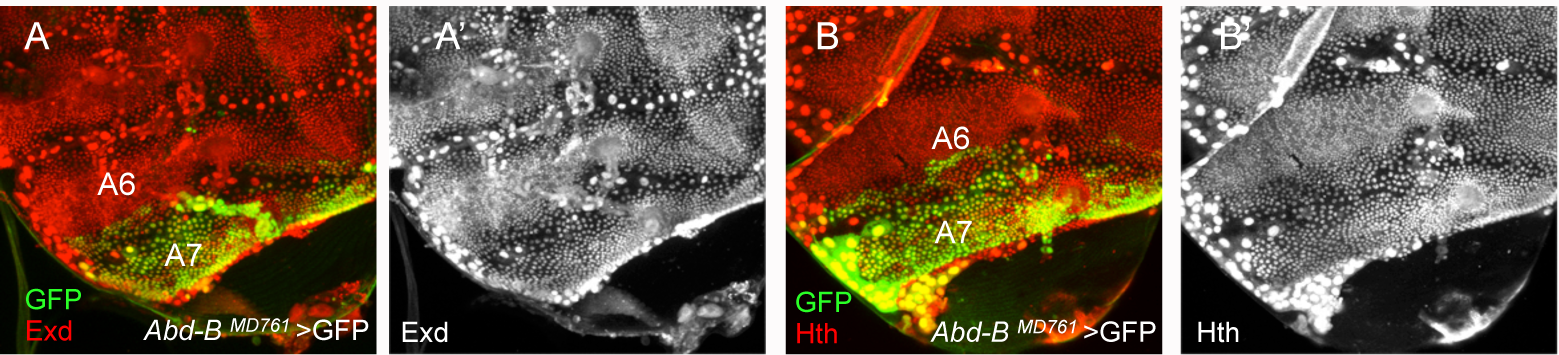

Supplement: S1 Fig — Expression of Exd (A, A’) and Hth (B, B’) in Abd-BMD761 UAS-GFP male pupa of about 28-30h APF, showing similar levels of expression of Exd and Hth in the A7 and A6 segments. Abd-BMD761 is a Gal4 line driving expression in the A7; see Methods and main text. (TIF) [file pgen.1011355.s001.tif]

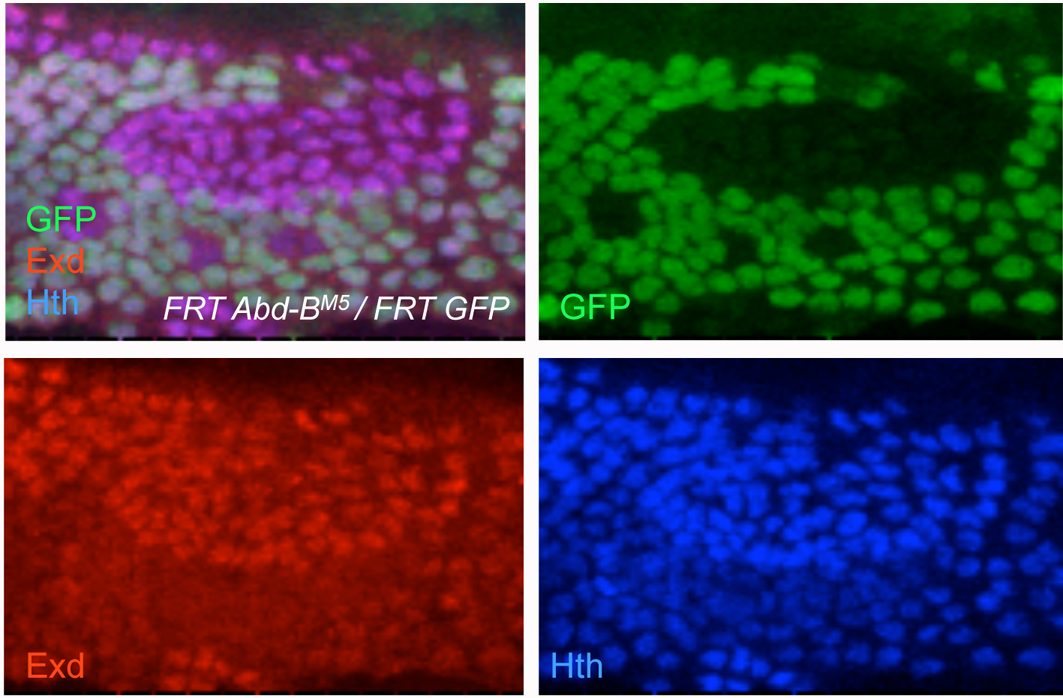

Supplement: S2 Fig — Big clone mutant for Abd-BM5 induced in the male A7 segment and marked by the absence of GFP, showing slightly increased levels of Exd (in red) and Hth (in blue) with respect to most adjacent cells. (TIF) [file pgen.1011355.s002.tif]

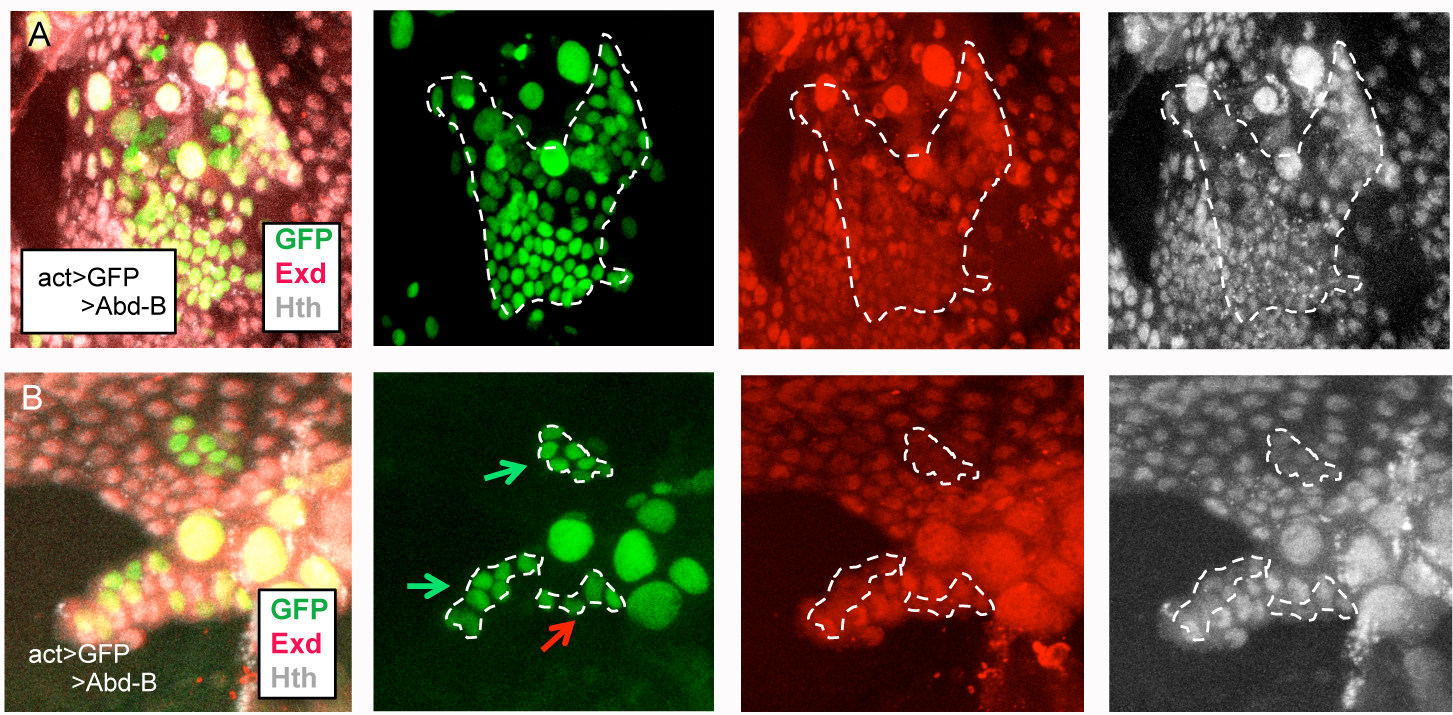

Supplement: S3 Fig — (A) Clone expressing Abd-B and marked with GFP in the A6. The expression of Exd (red) or Hth (grey) is not modified with respect to surrounding cells. (B) Clones expressing Abd-B and marked with GFP in the A7. The expression of Exd (red) or Hth (grey) is slightly reduced (except for one nucleus) with respect to surrounding cells in two clones (green arrows) but only in two nuclei in another clone (red arrow). (TIF) [file pgen.1011355.s003.tif]

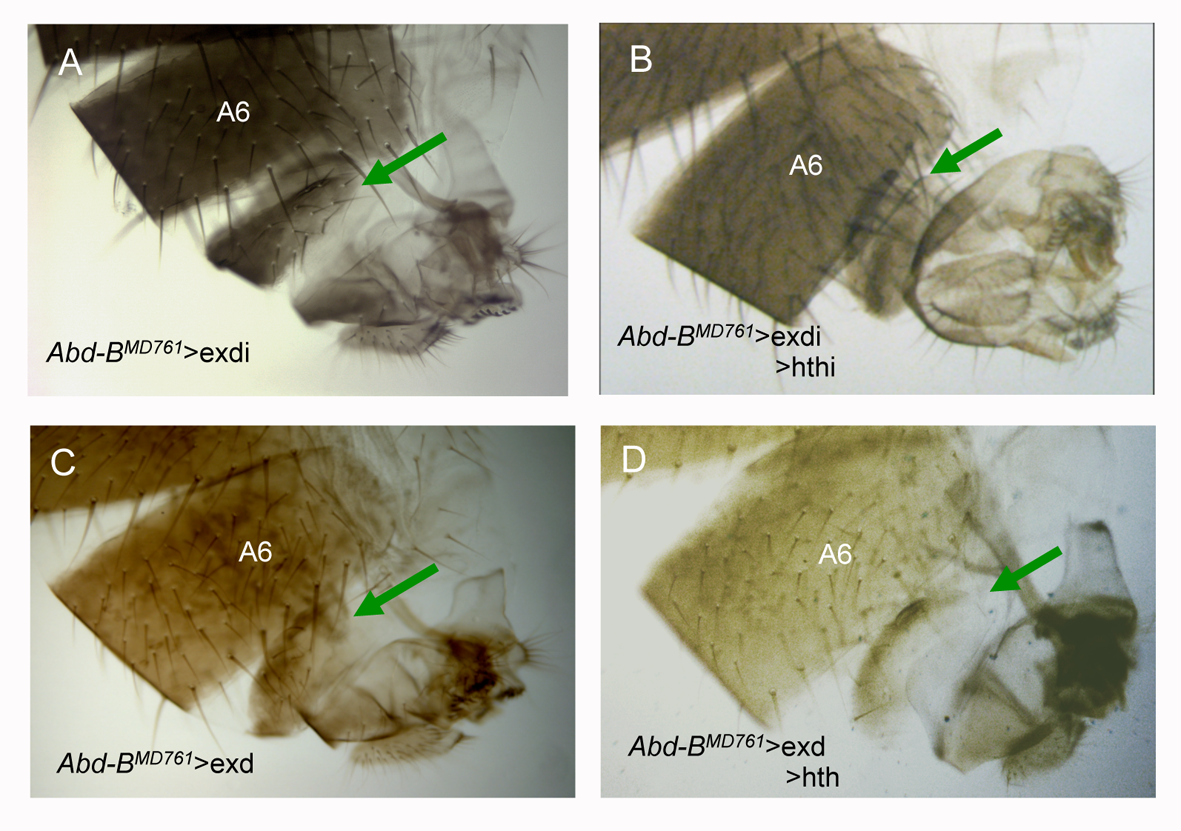

Supplement: S4 Fig — (A) The reduction of exd expression in the male A7 transforms it partially to A6, and this transformation is not increased by the simultaneous reduction of exd and hth expression in this segment (B). (C, D) The increase of either exd alone (C) or exd and hth (D) in the male A7 results in the development of a small segment of similar size in both genotypes. (TIF) [file pgen.1011355.s004.tif]

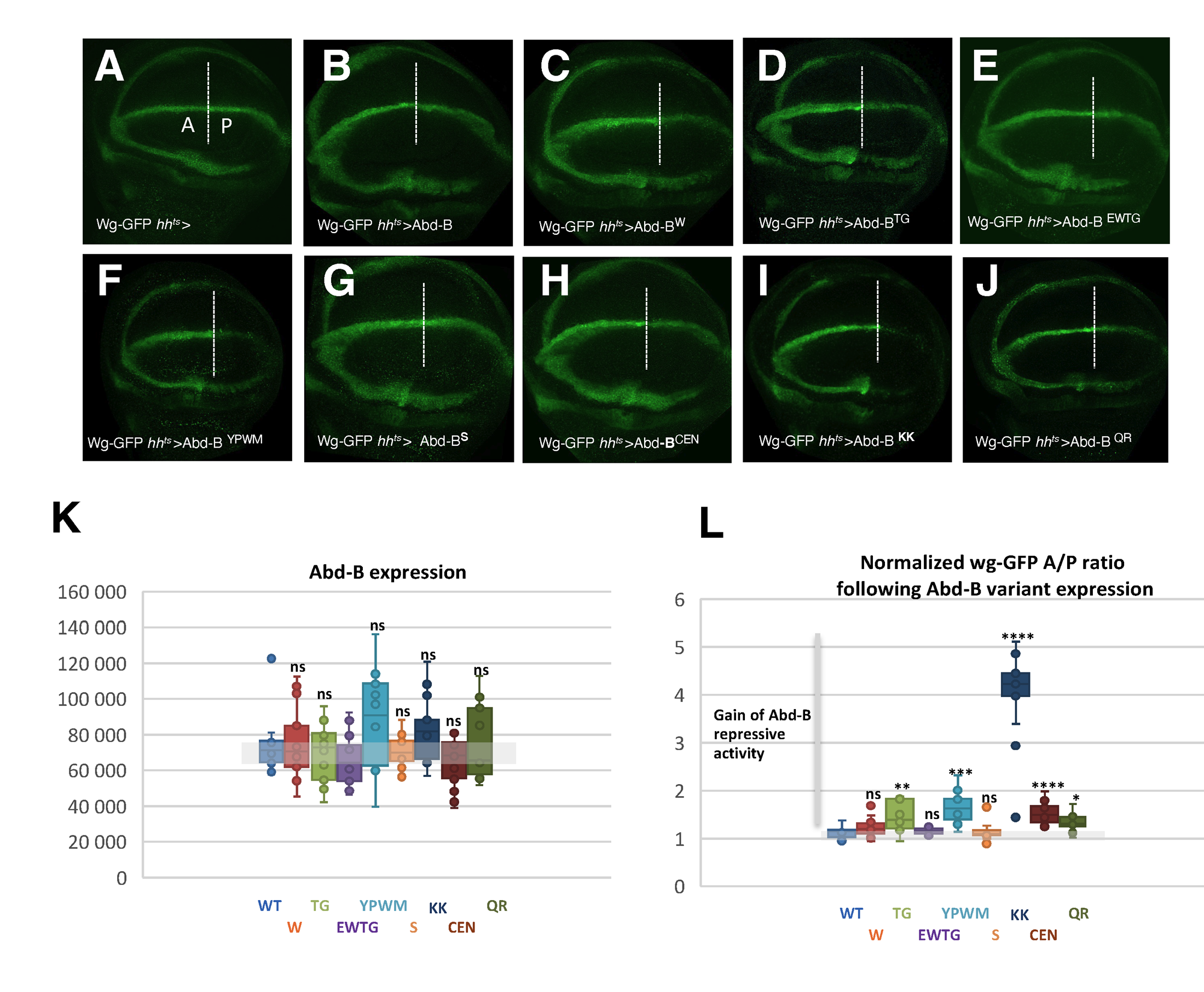

Supplement: S5 Fig — (A-J) The different panels show wg-GFP expression in wing discs of the hh-Gal4 tub-Gal80ts/+ genotype and of hh-Gal4 tub-Gal80ts driving expression of the different Abd-B proteins. In all cases the larvae were transferred in second or early third instar larvae from 18° to 29°C. In the wing pouch the wg-GFP line directs GFP expression, as wg, in two rings around the pouch and in a dorso-ventral band. (K) Quantification of Abd-B expression driven by hh-Gal4 in the posterior wing pouch. The wild type and Abd-B variant proteins do not show significant differences in expression levels (n = 10–15 for the wildtype Abd-B and all the variants). (L) Normalized wg-GFP expression following expression of Abd-B and Abd-B variants in the posterior wing pouch. Measurements were taken at the D/V boundary, both in the A (no Abd-B variant expression) and P (Abd-B variant expression) compartments (see methods); (n = 10–13 for the wildtype Abd-B and all the variants). The A/P ratio of wg-GFP expression is plotted and was normalized relative to the level of Abd-B and Abd-B protein variant expression (counts / ratio Abd-B variants/ Abd-B). Significant differences identify gain in Abd-B repressive activity for Abd-BTG, Abd-BYPWM, Abd-BKK, Abd-BCEN and Abd-BQR. (TIFF) [file pgen.1011355.s005.tiff]

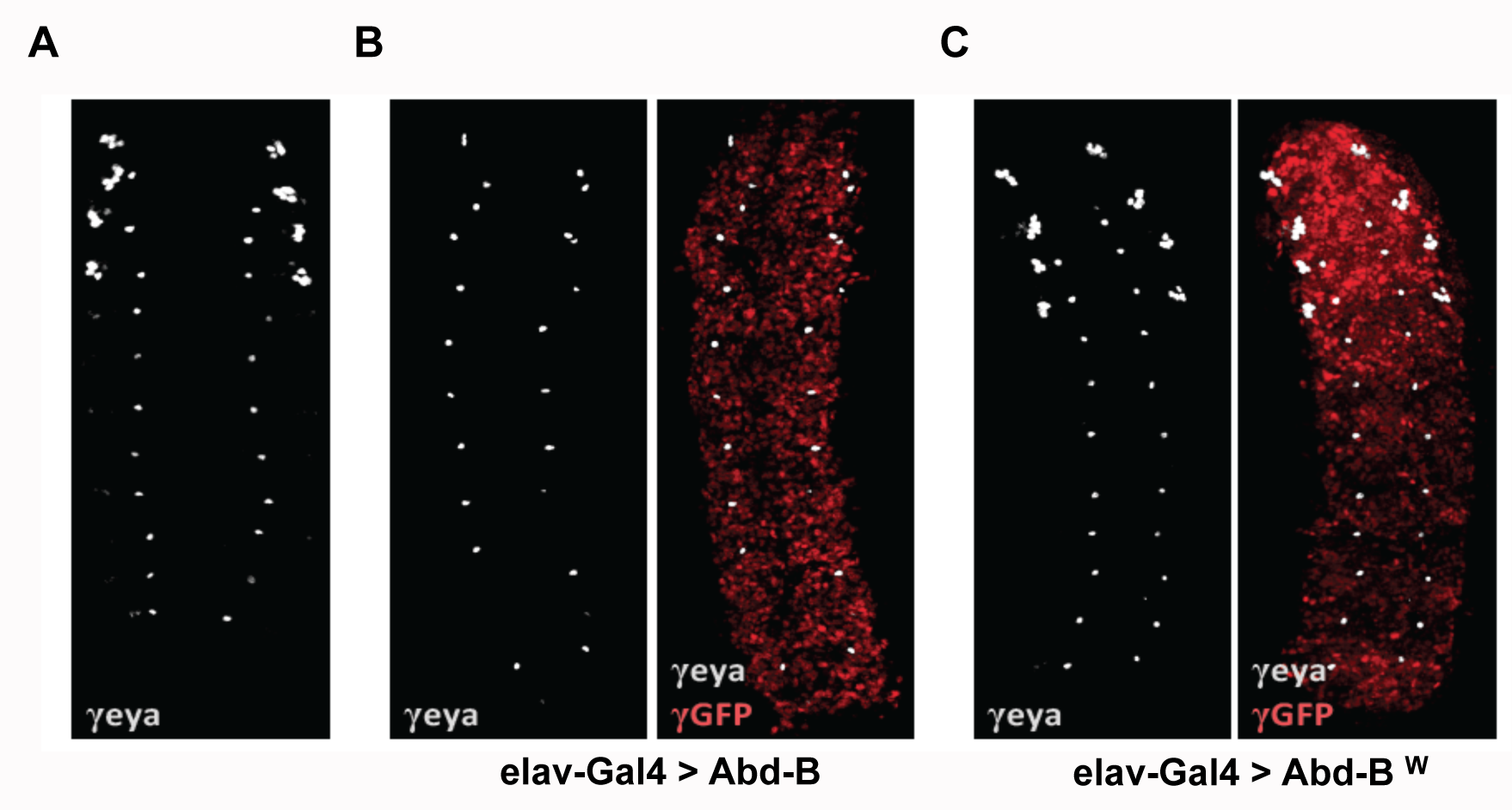

Supplement: S6 Fig — (A) Wildtype Eya expression. B). Forced elav-driven expression of Abd-B in the thorax results in the lack of thoracic specific Eya neurons. (C) Mutation of the W residue alleviates the repression in these neurons. (TIF) [file pgen.1011355.s006.tif]
