# Supplementary material for: Ambivalent partnership of the Drosophila posterior class Hox protein Abdominal-B with Extradenticle and Homothorax
Source: PLoS Genet. 2025 Jan 13;21(1):e1011355. doi: 10.1371/journal.pgen.1011355 (PMC11759358; doi:10.1371/journal.pgen.1011355)
Supplement: S1 Text — (DOCX) [file pgen.1011355.s008.docx]

Full genotypes of individuals used in each figure and panel

**Figures**

Figure 1: A, B- wildtype

C- hs-flp1.22;; FRT82B Abd-B^M5^/FRT82B ubi-GFP

D- pnr-Gal4 UAS-GFP/+

E- pnr-Gal4 UAS-GFP/UAS-Abd-BRNAi

F- hs-flp1.22; act>y+>Gal4 UAS-GFP/UAS-Abd-B (1.1)

G- pnr-Gal4 UAS-GFP/+

G- UAS-Abd-B (1.1)/+; pnr-Gal4 UAS-GFP/+

Figure 2: A- hs-flp1.22;; FRT82B hth^P2^/ FRT82B ubi-GFP

B- hs-flp1.22; act>y+>Gal4 UAS-GFP/+; UAS-hthRNAi/+

C- pnr-Gal4 UAS-GFP/+

C- UAS-exdRNAi/+; pnr-Gal4 UAS-GFP/+

C- pnr-Gal4 UAS-GFP/ UAS-hthRNAi

D- pnr-Gal4 UAS-GFP/ UAS-hth

G- hs-flp1.22; act>y+>Gal4 UAS-GFP/+; UAS-hth/+

Figure 3: A- wildtype

B- UAS-exdRNAi/+; pnr-Gal4 UAS-GFP/+

C- y w hs-flp1.22;; FRT82B hth^P2^/ FRT82B hs-CD2 M(3)i55 y^+^

D- pnr-Gal4 UAS-GFP/ UAS-hth

E- Abd-B^MD761^ UAS-GFP/+

F- Abd-B^MD761^ UAS-GFP/Abd-B^M1^

G- Abd-B^MD761^ UAS-GFP/UAS-hthRNAi

H- UAS-exdRNAi/+; Abd-B^MD761^ UAS-GFP/+

I- UAS-exdRNAi/+; Abd-B^MD761^ UAS-GFP/DpP5

J- Abd-B^MD761^ UAS-GFP/UAS-hth

K- UAS-exd; UAS-y^+^/+; Abd-B^MD761^ UAS-GFP/+

L- UAS-exd; UAS-Abd-B/+; Abd-B^MD761^ UAS-GFP/+

M- Abd-B^MD761^ tub-Gal80^ts^/Abd-B^M1^

N-UAS-Abd-B/UAS-GFP; Abd-B^MD761^ tub-Gal80^ts^/Abd-B^M1^

O- UAS-Abd-B/UAS-exdRNAi; Abd-B^MD761^ tub-Gal80^ts^/Abd-B^M1^

P- Abd-B^MD761^ tub-Gal80^ts^/Abd-B^M1^

Q- UAS-Abd-B (1.1)/UAS-y^+^; Abd-B^MD761^ tub-Gal80^ts^/Abd-B^M1^

R- UAS-Abd-B (1.1)/+; Abd-B^MD761^ tub-Gal80^ts^/Abd-B^M1^ UAS-hthRNAi

Figure 4: A- Abd-B^MD761^ UAS-GFP/+

A- Abd-B^MD761^ UAS-GFP/Abd-B^M1^

A- Abd-B^MD761^ UAS-GFP/UAS-exdRNAi

B- wg-GFP/UAS-y^+^; pnr-Gal4 tub-Gal80^ts^/UAS-cherry

B- wg-GFP/UAS-Abd-B; pnr-Gal4 tub-Gal80^ts^/UAS-y^+^

B- UAS-exd; wg-GFP/UAS-Abd-B; pnr-Gal4 tub-Gal80^ts^/+

Figure 5: C- *pnr*-Gal4 *tub*-Gal80^ts^ UAS-Abd-B::VC UAS-VN::Exd

D- *pnr*-Gal4 *tub*-Gal80^ts^ UAS-Abd-B^W^::VC UAS-VN::Exd

Figure 6: C, D, E- UAS-Abd-B (lines Abd-B^wt^, Abd-B^W^, Abd-B^TG^, Abd-B^EWTG^, Abd-B^S^, Abd-B^KK^, Abd-B^CEN^)/+; Abd-B^MD761^ tub-Gal80^ts^/Abd-B^M1^ and Abd-B^MD761^ tub-Gal80^ts^/Abd-B^M1^ UAS-Abd-B (lines Abd-B^YPWM^, Abd-B^QR^)

Figure 7: A- Abd-B^LDN^/+

B- Abd-B^LDN^/Abd-B^M1^

C- UAS-exdRNAi/+; Abd-B^LDN^/+

E, F- UAS-Abd-B (lines Abd-B^wt^, Abd-B^W^, Abd-B^TG^, Abd-B^EWTG^, Abd-B^S^, Abd-B^KK^, Abd-B^CEN^)/+; Abd-B^LDN^/Abd-B^M1^ and Abd-B^LDN^/Abd-B^M1^ UAS-Abd-B (lines Abd-B^YPWM^, Abd-B^QR^)

Figure 8: A- sca-Gal4 CycE lacZ/+

sca-Gal4 CycE lacZ/UAS AbdB

sca-Gal4 CycE lacZ/ UAS AbdB^W^

sca-Gal4 CycE lacZ/ UAS AbdB^QR^

**Supplementary Figures**

Supplementary Figure 1:

A- Abd-B^MD761^ UAS-GFP/+

B- Abd-B^MD761^ UAS-GFP/+

Supplementary Figure 2:

A- hs-flp1.22;; FRT82B Abd-B^M5^/FRT82B ubi-GFP

Supplementary Figure 3:

A, B- hs-flp1.22; act>y+>Gal4 UAS-GFP/UAS-Abd-B (1.1)

Supplementary Figure 4:

A- UAS-exdRNAi/+; Abd-B^MD761^ UAS-GFP/+

B- UAS-exdRNAi/+; Abd-B^MD761^ tub-Gal80^ts^ /UAS-hthRNAi

C- UAS-exd;; Abd-B^MD761^ UAS-GFP/+

D- UAS-exd;; Abd-B^MD761^ UAS-GFP/UAS-hth

Supplementary Figure 5:

A- wg-GFP/+; hh-Gal4 tub-Gal80^ts^/+

B-J- wg-GFP/UAS-Abd-B (lines Abd-B^wt^, Abd-B^W^, Abd-B^TG^, Abd-B^EWTG^, Abd-B^S^, Abd-B^KK^, Abd-B^CEN^); hh-Gal4 tub-Gal80^ts^/+ and - wg-GFP/+; hh-Gal4 tub-Gal80^ts^/ UAS-Abd-B (lines Abd-B^YPWM^, Abd-B^QR^)

Supplementary Figure 6:

A- wt

B- elav-Gal4/+ or Y; UAS-GFP UAS-Abd-B

C- elav-Gal4/+ or Y; UAS-GFP UAS-Abd-B^W^
