# Supplementary material for: Ambivalent partnership of the Drosophila posterior class Hox protein Abdominal-B with Extradenticle and Homothorax
Source: PLoS Genet. 2025 Jan 13;21(1):e1011355. doi: 10.1371/journal.pgen.1011355 (PMC11759358; doi:10.1371/journal.pgen.1011355)
Supplement: Attachment — Submitted filename: Response to reviewers.docx [file pgen.1011355.s009.docx]

Dear Editor,

We wish to thank the referees for their constructive feedback on our manuscript. We show below our detailed answer to their comments and requests (in italics and bold). The changes we have made include experimental additions leading to two new Supplementary Figures (Suppl. Figures 3 and 4), elimination of a previous Suppl. Fig. 3 (as requested by Referee 2), and several changes in the text, including an extensive rewriting of the Discussion section. All changes can be followed in the marked-up copy of the revised manuscript.

We summarize here the main changes:

1- We have simultaneously inactivated *exd* and *hth* in the male A7 segment and also activated both in the same segment, as requested by Referee 1. This is shown in the new Supplementary Fig. 4.

2- We have analyzed clones expressing Abd-B in the male posterior abdominal segments, as suggested by Referee 2. The results are shown in the new Supplementary Fig. 3.

3- We have quantitated the *Co-IP* experiments, as suggested by Referee 1. The results are shown in Fig. 5.

4- We have re-written the Discussion section, trying to make it simpler, avoiding recapping conclusions already formulated in the Results section, as suggested by Referee 3.

We feel we have addressed or discussed all points raised by the Referees, what has much improved the manuscript.

We hope you will find this revised manuscript suited for publication.

With best regards,

Ernesto Sánchez-Herrero

**Responses to Referee’s comments and requests are directly inserted in the text of their reviews.**

***REFEREE #1:***

*How proteins have evolved across animal kingdom with diverse functions, most generally specifying segment-specific developmental pathways along with the antero-posterior axis. Interestingly, while Hox proteins themselves have not evolved much, evolution of diversity in body plan in closely related species is attributed to variations in the way Hox proteins interact with their co-factors and/or changes in regulatory/coding sequences of their targets.

This MS by - Curt et al - based on the work done in the laboratories of Graba and Sanchez-Herrero - is an example of how function of AdbdB to suppress the development of 7th abdominal segment. They have shown that in this context, Exd/Hth interact with AbdB differently than in other regions during development. They have shown that expression patterns/levels of Exd/Hth is inversely related to that of AbdB, still the three are required together for their biological function.

The study is well planned, experiments are well executed and results are consistent with their hypothesis and interpretations.

This MS is also an example of fine genetics experiments that authors have carried out.

I recommend its publications in Genetics, while have following comments/suggestions for its further improvement.*
 **1. *The work is incomplete without showing how Ex/Hth levels are maintained.***

Our work has concentrated in the relationship of Abd-B with Exd/Hth. Cross regulations are described in Figs. 1 and 2 . We did not address how the expression of Exd/Hth, ubiquitously distributed in the pupal abdomen, in histoblasts and larval epidermal cells, is maintained. Although of interest, we feel this is not within the scope of the present study.

**2. *What happens when both Hth and Exd are over-expressed or knocked-down?***

We have done these experiments. The combined expression of *exd* and *hth* does not make a bigger A7 than the single expression of either of the two and the reduction in expression of both genes does not result in a stronger effect than the inactivation of either. We have included these results as Supplementary Figure 4.

**3. *IP experiments suggest Hth can bind to AbdB function independent of Exd. A6 to A5 (more anterior segment) transformations are seen in both over- and loss-of-expression of Hth. Is it possible that when Hth is over-expressed, Exd becomes a limiting factor making hth-AbdB complex dominant negative to Hth/Exd/AbdB complex?***

This is an interesting idea. We have added this hypothesis in the section **“**Abd-B associates with Exd/Hth in the pupal abdomen independently of the generic HX interaction motifs”.

**4. *The Co-IP experimental results should be quantitated against input levels.***

This has been done and it is shown in Fig. 5. More specifically, we now present in panel 5A the Elution/Input ratio for Abd-B and Exd co-IP (not for Hth, which is the bait in the experiment), and in panel 5B we calculated the loss of Abd-B and Exd binding to the Hth bait induced by the tryptophan mutation. While proceeding for quantification, we changed a few panels in the figure, as for some of them we could not retrieve the full original blots. Experiments were performed multiple times and were consistent: these changes thus do not alter any of the previous conclusions. This has been done and it is shown in Fig. 5.

***5. Figures legends need to be re-written. For example,
4 (B) Expression of wg-GFP in the A4 segment of males of the following genotypes: wg-GFP/UAS-y+; pnr-Gal4 tub-Gal80ts/UAS-cherry (n=10), wg-GFP/UAS-Abd-B; pnr-Gal4 tub- Gal80ts/UAS-y+ (n=10) and UAS-exd; wg-GFP/UAS-Abd-B; pnr-Gal4 tub-Gal80ts/+ (n=12), all shifted from 18ºC to 29ºC in third larval stage, showing large coincident expression of GFP
55 and cherry in the control, partial repression of wg-GFP when Abd-B is expressed in the pnr domain and partial suppression of this repression if exd is also expressed in this mutant combination.
Very confusing sentence. I guess, authors want to say that when Abd-B is over-expressed, Wg expression is suppressed and this is reversed/rescued when Exd is also over-expressed. This has not come out clearly. All figure legends may be thoroughly edited to make the sentences simpler and clear.***

We have modified this figure legend and checked the other figure legends as well.

**REFEREE 2**

*This is an interesting and complex paper in which the levels of expression as well as the function of Abd-B in the interaction with Exd-Hth is analysed in Drosophila melanogaster. In the past, two publications in PLoS genetics ( Sambrani et al. 2103; Rivas et al. 2013), demonstrated that, contrary to other Hox genes, the Abd-B Hox protein has an antagonistic relationship with the Hox cofactors Exd-Hth during development. In the current study Curt and collaborators extend this work and explore the interaction of Abd-B with the hox cofactors in further tissues. They show that in different tissues the Abd-B Exd-Hth interaction may be antagonistic while in others the interaction is synergistic as it is accepted for more anterior Hox proteins. Besides the complexity that such tissue/organ differences on Abd-B function reveal, the authors show that the cross regulation that Abd-B can have on Hth-Exd levels of expression and vice versa add an extra level of complexity to any study of this kind. The paper is very thorough, analysing genetic interactions, protein-protein biochemical interactions by Co-IP, protein complex DNA interactions by EMSA and in vivo protein interactions using BiFC analysis. The authors also mutagenize the Abd-B homeodomain and flanking regions to study how different amino acids contribute to the synergistic or antagonistic Abd-B interactions.
The authors conclude that the relationship of Abd-B with the Hox cofactors can be either antagonistic, synergistic or dispensable (probably when the Abd-B protein acts as a monomer or with unknown co-factors) depending on the organ where it functions.

Specific comments*
***- The authors should credit in the abstract previous work showing Abd-B Exd-Hth antagonism.***

We have modified the Abstract accordingly.

***- Except when a direct target is analysed, as is the case for CyclinE or Dll enhancers, the phenotypes obtained are not necessarily due to a single interaction of the Abd-B-Exd-Hth complex on one target but a sum of several. This may confound the interpretations as some of the observed effects can also be exerted independently by Abd-B acting as a monomer or by Exd-Hth controlling Hox independent functions (for example, Engrailed function has been shown to be affected by Exd-Hth and other unknown proteins may also be interacting). This should be discussed*.**

We cannot exclude these possibilities, which may indeed contribute to the phenotypes studied. This is now stated in the Discussion section **“**Synergism versus antagonism: mechanisms and Hox class specificity”.

***- The effect on pigmentation caused by ectopic Exd-Hth (Fig 3D and pg27 Discussion) is described as an antagonistic competition with Abd-B. In my opinion this is not that clear. Ectopic Exd-Hth expression also affects pigmentation in segments where Abd-B is not expressed, and the repression of pigmentation in the A4-5 abdominal segments does not agree with lack of anterior Hox expression. The lack of pigmentation could be a Hox independent effect. The formation of ectopic trichomes seems like a more convincing antagonistic phenotype, although the authors should present in Fig 3 close up images that show the trichomes more clearly*.**

It is known that reduction of *exd* or *hth* expression results in dark pigmentation in anterior abdominal segments. We have not found in the literature, however, an effect on pigmentation of an excess of *exd* or *hth*; rather, the increased expression of *exd* is reported to make no effect in the adult (Rauskolb et al., 1995, Development). *Abd-B* is known to regulate pigmentation (Jeong et al., 2006, Cell) and therefore, if an excess of *hth* or *exd* reduces Abd-B activity, it is expected that this results in reduction of pigmentation in the A5 and A6 abdominal segments (to resemble now anterior abdominal segments). This agrees with previous interpretation of the phenotype observed in the abdomen after high increase of *hth* with the Ga4/UAS system, the same experiment we have done (Ryoo et al., 1999, Development). We cannot rule out, nevertheless, that *exd*/*hth* also contribute to pigmentation independently of *Abd-B*, so we have modified the sentence, as suggested by the referee.

Close-up images to show more clearly the trichomes are in the panels A’-D’ of Fig. 3.

***- In the text Pg 17 it says that the Abd-B wild type and Abd-B w protein rescuing ability of A7 suppression is very similar, but in Fig 6E it marks the interaction as highly significant (p<0.0005). Similarly, in pg 18 it says the Tryptophan residue is dispensable for A7 suppression. I believe these incongruences may be due to a mistake on Fig 6E as it seems that the n.s. and **** symbols are displaced one column to the left (and as a result there is a symbol missing over the TG column). If this was not a mistake on the figure, the text should be modified as the difference is highly significant.***

This was a mistake that has been corrected. Thanks to the referee for pointing this to us.

***- in pg 18 the authors talk about the rescue of the vaginal teeth by rescuing with different UAS-Abd-B variants the lack of genitalia present in Abd-B LDN/Abd-B M1 females. As to form vaginal teeth it is necessary to have a genital disc, it is unclear why the authors insist on talking exclusively about restoring the vaginal teeth function (5th line on page 19) when what they do is to rescue the genital disc. Counting the vaginal teeth is a good way to quantify the genital disc rescue, but there is no reason to say that the proteins are specifically rescuing the vaginal teeth. This also applies to Fig.9 where the antagonism is on genital disc formation and not on vaginal teeth formation. Similarly, in 5th line pg 20, pg 28 last line and pg 30 5th line, it should talk about genitalia formation rather than vaginal teeth development*.**

The referee is right. We have rewritten the text in the lines indicated regarding female genital disc formation and vaginal teeth development.

***- In pg 19-20: The fact that KK mutations and to a lesser extent the TG and CEN variants have increased wg-GFP repressive activity in the wing pouch where Hth is not expressed indicates that there is an unknown factor modulating Abd-B function that in other tissues can or cannot be modulating the Hth-Exd Abd-B interaction.***

We have included this idea when describing the repression of wg-GFP in the Results section.

**-*Pg 9 “Thus, while wild type expression of Abd-B in A7 (…) in A3-A5 does.”
Why is A6 omitted?***

This is related to the question of the referee below about *Abd-B*-expressing clones in the A6 and A7. With this set of experiments, we aimed at observing the effect of *Abd-B* expression in segments devoided of Abd-B (A3, A4) or with very low levels of this protein (A5), both in clonal and in *pnr*-driven gain of function experiments. That is why we initially did not address the effect of increasing *Abd-B* expression in A6 and A7, where Abd-B is highly expressed. After the request of the referee we have looked to *Abd-B*-expressing clones in these two segments, finding no effect on *exd* and *hth* expression or reduced signal of both. The text has been modified, making the aim of the experiments clearer. Also, the new data are now included in the text and in Supplementary Fig. 3.

**- *Pg 12 describe what genes are affected by DpP5. Only Abd-B or more Bx-C elements?***

DpP5 is a tandem duplication for the whole BX-C (Duncan and Lewis, 1982). We have indicated in the Results and Material and Methods sections that it duplicates the three genes of the BX-C.

***- In pg 17 the CEN mutation is said to be KRKP>CRTQ but in Fig. panel 6B it is shown as GRTQ. Which one is correct?***

This has been changed. GRTQ is correct. Thanks to the referee for pointing to us this mistake.

***-Pg 20. The authors should show the outcome of over expressing Exd-Hth in the CNS to conclude there is pure synergism in the CNS. The situation could be similar to what they observed for A7 repression.***

The synergistic action of Exd/Hth and the Hox protein Abd-A was previously demonstrated, showing cooperative DNA binding and induced *cycE* repression when Abd-A expression is ectopic in the thoracic segments (Kannan et al., Mechanism of Development, 2010). This may have been missed as the reference we indicated was not the proper one, which is corrected in this revised version. In short, the rational of the experiment is that given that Exd and Hth are already expressed in the thorax (and abdomen), the limiting factor for *cycE* repression is the presence of the Hox protein. This was previously shown for Abd-A, and here we show that this is also the case for Abd-B. Given that we also see cooperative binding on the *cycE* enhancer, we conclude, as previously done for Abd-A, that Abd-B synergizes with Exd/Hth for *cycE* repression. We do not think that increasing Exd/Hth would modify this synergic effect. The text in the result section has been modified, to include the proper reference, and to clarify the rational of the experiment.

***- Fig.1F should present the effect of clones or pnr-Gal4 expression in A6-A7 to show if these segments behave differently.***

See answer above.

*Minor comments:*

***- Drosophila in italics all over the text.***

This has been changed.

**-*Pg 9. If at later stages the Hth increase is seen both in experimental and control experiments (Sup. Fig.3), is this relevant for the paper? I would consider deleting the sentence starting with: “At later stages (about 34-38h…)“.***

We have removed the sentence and the Supplementary Figure.

**- *Pg 20: Modify title. Mutations are not required. Especially in this case. It is the W a.a. that is required for Abd-B CNS function*.**

This has been modified.

**- *Lettering in Fig. 8 has to be improved, the resolution is not good*.**

We have increased one point the size of some genotypes, to try to make it clearer.

**-*Fig 6 legend: “…most conserved domains…” specify where: in insects, arthropods or also in vertebrates?***

Web logo was obtained using sequences for the following AbdB sequences (Drosophila (AAA84402), Tribolium (AAF36721.1), Anophela (XM311628), Sacculine (AAQ49317.1), Folsomia (AAK52499.1) and human (BCO10023). We have included this information in the legend to Fig. 6.

***- Pg. 25.* *“Consistently, ectopic Abd-B (…) exd expression.” Does this refer to the wing, the histoblasts or both?***

This referred to histoblasts. The paragraph was re-written.

***- Pg. 25. “This highlights the importance (…) proposed for Ubx …” An earlier paper should also be cited: Irvine et al. 1993 Development 117, 387-99.***

The reference has been included.

**- *Pg 31 “as about 25% longer”. Shouldn’t it say shorter or faster?***

We have replaced longer by faster.

**REFEREE 3**

*This manuscript, titled “Ambivalent partnership of the Drosophila posterior class Hox protein Abdominal-B with the Extradenticle and Homothorax cofactors “ by Curt, et al defines interactions between Abd-B, Hth and Exd in different contexts in Drosophila development. A careful examination of the wild-type expression of exc, hth and Abd-B in A7 is followed by a close examination of cross-regulation by these factors on one another. Both LOF and GOF of hth/exd were shown to lead to anterior transformations of A6 in males and mis-regulation of Wg. CoIP experiments examine interactions between these proteins in various contexts. Finally, specific point mutations in Abd-B in the YWPM-anologous region and C-terminal of the homeodomain shows context-dependent activities of many residues on the phenotype in several tissues in which Abd-B plays a role. This study has been meticulously conducted, is presented well overall and this reviewer applauds the breadth and depth of the study, genetically and mechanistically. It adds significantly to our understanding of AbdB Hox function with Exd and Hth in Drosophila. It also brings new insight to the mechanisms potentially used by all posterior Hox genes in other organisms.*

***One suggestion for author consideration: the discussion is nearly as complex as the results. There may be an opportunity to do less recapitulating of the results and more discussion of implications (which is already there but might be expanded). This reviewer, for instance, is intrigued by the idea that the context-dependent activities could be related to specific expression of Hox, Exd or Hth as well as other co-factors in tissues/organs as they develop past segments, an idea supported by some of the data presented.***

We have rewritten the Discussion, trying to make it simpler and clearer, as the referee suggested.

***Also for author consideration: Would “Context-dependent” be better word choice in title than “Ambivalent” for title – seems anthropomorphic to this reviewer? And perhaps drop the word ‘cofactors’ as, at least in some of their roles, it seems they aren’t acting as cofactors?***

We would like to keep the word “ambivalent”, which seems to us more suitable than “context-dependent”.

“Context dependent”, which would also be right, only refers to the point that the effects are different. “Ambivalent” in our view it implies opposing effects, which is not conveyed by “context dependent”.

We have changed the word cofactors by partners.

*Minor comments:*

***Page 8: for non-Drosophilists, can the authors more carefully describe ‘extruded’ for the 7th abdominal segment. It is formed but lost from pupae?***

The referee is right, that is what it happens and we have explained it now in the text.

***Likewise, Figure 1 and 2 are presented in such a way that a non-Drosophila geneticist can pretty easily understand the genetics shown. This is more difficult in many following figures. The authors could consider providing a bit of additional information in the figures to help with this aspect.***

We have tried to add information to the Figures whenever possible, for example in Fig. 4, but in other we have found that adding more information could make them more confusing.
